# Randomized, Double-Blind, Placebo-Controlled Study of the Safety, Tolerability, and Clinical Effect of Danirixin in Adults With Acute, Uncomplicated Influenza

**DOI:** 10.1093/ofid/ofz072

**Published:** 2019-04-22

**Authors:** Grace Roberts, Shuguang Chen, Phillip Yates, Anuradha Madan, Jill Walker, Michael L Washburn, Andrew J Peat, Gary Soucie, Edward Kerwin, Sumita Roy-Ghanta

**Affiliations:** 1 GlaxoSmithKline, Research Triangle Park, North Carolina; 2 GlaxoSmithKline, Upper Providence, Pennsylvania; 3 GlaxoSmithKline, Stevenage, Herts, United Kingdom; 4 Elite Clinical Trials, Blackfoot, Idaho; 5 Clinical Research Institute of Southern Oregon, Inc., Medford

**Keywords:** CXC chemokine receptor 2 (CXCR2) antagonist, danirixin (DNX), influenza, outpatient, safety

## Abstract

**Background:**

Danirixin (DNX), a selective and reversible CXC chemokine receptor 2 antagonist, inhibits neutrophil transmigration and activation. This study assessed the safety, tolerability, and clinical effect of DNX with and without oseltamivir (OSV) in adults with acute, uncomplicated influenza.

**Methods:**

This was a placebo-controlled, double-blind, Phase IIa study. Participants (18–64 years) with influenza-like symptoms (onset ≤48 hours) and positive influenza rapid antigen test were randomized 2:1:2:1 to DNX, placebo, DNX+OSV, or OSV (75 mg each, administered twice daily for 5 days) and followed for 28 days. Primary endpoints included frequency of adverse events (AEs) and serious AEs (SAEs). The effect of DNX on virologic response and clinical effect on influenza symptoms were secondary endpoints.

**Results:**

A total of 45 participants were enrolled, 35 of whom were confirmed influenza positive by polymerase chain reaction analysis. The highest incidence of AEs was in the placebo group (4 of 7, 57%), followed by the DNX+OSV (7 of 16, 44%), DNX (3 of 15, 20%), and OSV (0 of 7, 0%) groups. One SAE (T-wave abnormality) was reported in the DNX group (unrelated to treatment). No differences in viral load assessments were observed among treatment groups.

**Conclusions:**

Danirixin treatment was well tolerated and did not impede viral clearance.

Globally, between 250 000 and 650 000 deaths are associated with influenza respiratory diseases annually [1], despite the availability of vaccinations and direct-acting antiviral (DAA) drugs [2–7]. To be effective, currently available DAA drugs need to be given early in the course of disease [3, 4, 6, 8]. This suggests that exploration of novel therapeutic approaches, including mechanisms targeting later-stage disease processes to widen the window for effective intervention, is warranted to improve the treatment of seasonal and pandemic influenza.

Emerging preclinical and clinical data point to a pathogenic role for neutrophils in severe influenza infections [9, 10]. Neutrophils are among the first immune cells to infiltrate a site of infection to protect against pathogens; however, an excessive response has been demonstrated to cause lung damage through their release of tissue-destructive enzymes, reactive oxygen species, and extracellular traps [11]. Levels of pulmonary neutrophils and their associated chemokines, such as interleukin-8 (IL-8), are correlated with clinical symptom severity of influenza infection in humans [12–14], and aberrant immune response has been implicated in fatal cases of influenza [15, 16], suggesting a correlation between disease severity and the CXC chemokine receptor 2 (CXCR2)-IL-8 axis. Results from preclinical studies, using a top down systems approach, indicate that an excessive proinflammatory neutrophil response contributed to the lethal outcome of the infection. Therapeutic attenuation of neutrophil infiltration into the lung after influenza infection in mice improved lung integrity and survival, without increasing viral load [9, 17].

Danirixin (DNX) represents a novel treatment paradigm: it is a selective and reversible CXCR2 antagonist that inhibits neutrophil transmigration and activation at sites of inflammation [18]. Danirixin is currently being developed as a potential anti-inflammatory agent for the treatment of severe viral lower respiratory tract infections; it can be administered alone or as an adjuvant to an antiviral therapy. Preclinical studies have shown that therapeutic treatment of influenza infected mice with a CXCR2 antagonist in combination with the neuraminidase inhibitor oseltamivir (OSV) reduced pulmonary neutrophil levels and indicated improvements in overall clinical outcomes relative to animals receiving OSV monotherapy [19]. Furthermore, in a first-in-human study in healthy adults, dose-dependent inhibition of ex vivo agonist-induced neutrophil activation was observed after single and repeated once-daily oral administration of DNX [20].

The objective of this clinical study was to characterize the safety, tolerability, and clinical efficacy of DNX with and without OSV in otherwise healthy adults with acute, uncomplicated influenza, the results of which were used to progress DNX development into patients hospitalized with influenza treated with intravenous DNX [21].

## METHODS

### Study Design

**Figure 1.**
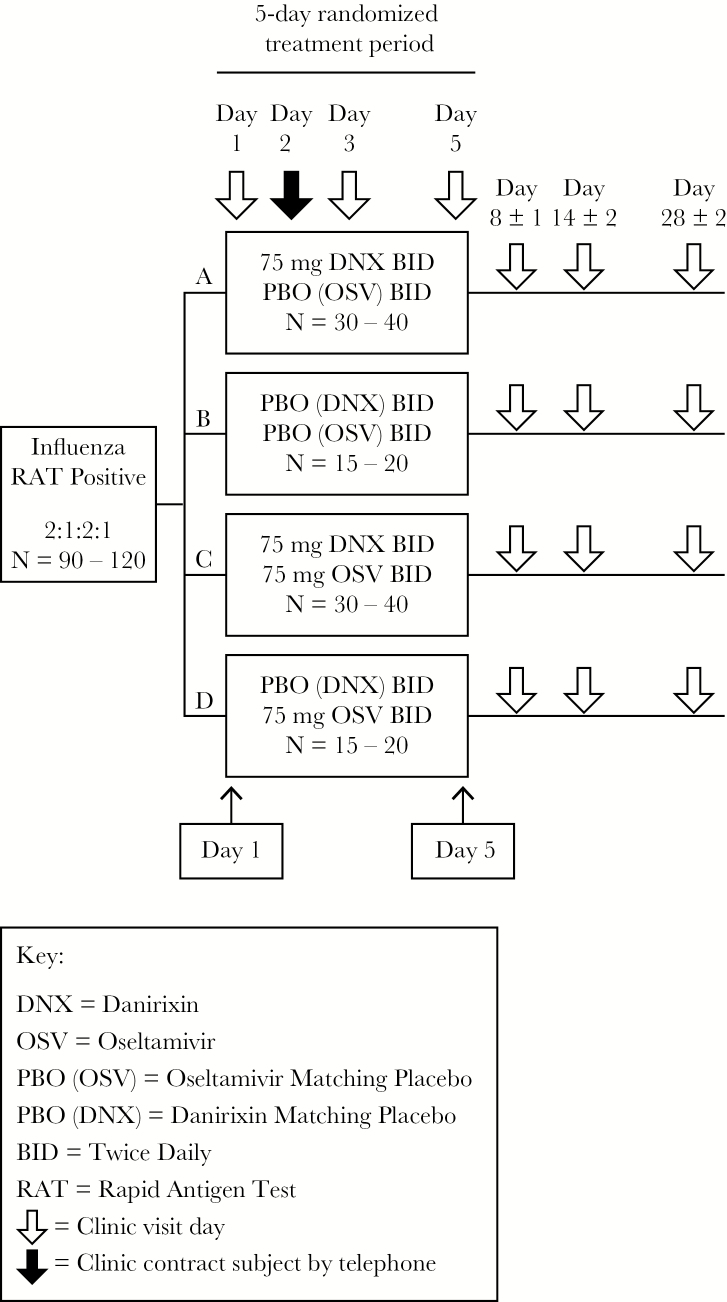
Study design. BID, twice daily; DNX, danirixin; OSV, oseltamivir; PBO, placebo; RAT, rapid antigen test.

This was an exploratory, Phase IIa, randomized, double-blind, placebo-controlled, 4-arm outpatient study (NCT02469298; GlaxoSmithKline [GSK] study number: 201682; initiation date: June 1, 2015; completion date: April 25, 2016) evaluating the safety, tolerability, and clinical effect of DNX with or without OSV, in otherwise healthy adults with laboratory-confirmed influenza infection (Figure 1). Patients were recruited in Australia, South Africa, and the United States at outpatient, primary care clinics, and the study period encompassed 1 influenza season in the southern hemisphere (June 2015–October 2015) followed by 1 season in the northern hemisphere (January 2016–April 2016). Details of participating centers are in Supplemental Table 1.

The study was conducted under an open Investigational New Drug Application (United States) and approved by the Therapeutic Goods Administration (Australia) and the Medicines Control Council (South Africa) and relevant ethics committees (Bellberry Human Research Ethics Committee, Australia; Pharma Ethics, South Africa; Quorum Review Inc. and Western Institutional Review Board, United States). The study was conducted in accordance with the International Conference for Harmonisation for Good Clinical Practice and the principles of the Declaration of Helsinki [22, 23] and was monitored by an Internal Safety Review Committee. All participants provided written, informed consent before study participation.

### Participants

Participants included in this study were males and females, aged 18–64 years, with a body weight of >60 kg (males) and >45 kg (females) and a body mass index of 19–35 kg/m^2^. Eligible participants, with a positive influenza virus rapid antigen test ([RAT] as assessed using the Sofia Influenza A+B Fluorescent Immunoassay), experienced the onset of influenza-like symptoms within 48 hours before enrollment or had a history of feeling feverish within 24 hours before the screening visit. The onset of symptoms was defined as the time when the participant’s temperature was first measured as elevated (≥38.0°C, ≥100.4°F) or the time when the participant first experienced at least 1 respiratory symptom (cough, sore throat, or nasal congestion) and at least 1 systemic symptom (headache, body aches and pains, or fatigue). A complete list of all inclusion criteria is provided in the Supplementary Material.

Participants at high risk of complications from influenza infection (according to the World Health Organization Guidelines for Pharmacological Management of Pandemic Influenza A [H1N1] and other influenza viruses) were excluded from the study. Participants at high risk included those who had chronic obstructive pulmonary disease, chronic cardiac disease, metabolic disorders, chronic renal disease, or hemoglobinopathies/immunosuppression, had taken an approved or investigational antiinfluenza medication within the last 4 weeks before enrollment, received the live-attenuated influenza virus vaccine within 21 days before the study, or if they required treatment with an influenza antiviral that was considered essential. Pregnant females were excluded from this study. A complete list of all exclusion criteria is provided in the Supplementary Material.

### Randomization and Blinding

Participants were randomized 2:1:2:1 to receive 75 mg of DNX, placebo, 75 mg of DNX plus 75 mg of OSV, or 75 mg of OSV twice daily, for a total of 10 doses over 5 days. Danirixin was administered in tablet form, and OSV was administered in capsule form; therefore, a double-dummy technique was used to maintain treatment blinding. Investigators and treating physicians were blinded to treatment allocation; in the case of an emergency or in the event of a serious medical condition, the investigator or treating physician could unblind a participant’s treatment assignment.

### Endpoints and Assessments

Participants were assessed on days 1, 3, 5, 8, 14, and 28. In addition, they were to complete the Influenza Intensity and Impact Questionnaire (Flu-iiQ) using an electronic diary twice a day until at least day 14. The Flu-iiQ is a validated questionnaire developed to document patient-reported outcomes in influenza [24].

The primary endpoints were as follows: the frequency of adverse events (AEs) and serious AEs (SAEs); changes in clinical laboratory evaluations, vital signs, and electrocardiogram (ECG) parameters; and the frequency of disease-related events (DREs) of interest (otitis media, sinusitis, bronchitis, and pneumonia) and associated antibiotic use.

The secondary clinical effect endpoints included the following: time to resolution of fever and proportion of afebrile participants (oral temperature ≤37.2°C, ≤99.0°F); incidence of hospital admissions due to influenza infection; change over time in influenza viral load (as measured by real-time quantitative reverse-transcription polymerase chain reaction [qRT-PCR] from nasopharyngeal swabs); and the percentage of participants with no detectable influenza virus at various time points. Time to resolution of symptoms, as measured by the Flu-iiQ questionnaire [24], was assessed as an exploratory endpoint. Biomarkers characterizing immune responses and target engagement were also measured as an exploratory endpoint and will be reported separately.

### Statistical Analysis

The target enrollment of 90–120 participants was predominantly based on feasibility of enrollment. No formal hypotheses were to be tested. The study was designed to characterize the safety of DNX with and without OSV. The safety population comprised all randomized participants who received ≥1 dose of study treatment. The influenza-positive population (IPP) comprised all randomized participants who received ≥1 dose of study treatment with proven influenza infection (positive RAT and positive influenza by qRT-PCR, or quantitative viral culture [qVC] at any time point). The IPP was used to assess clinical effect and for virology analyses.

Safety and virology data were analyzed using descriptive statistics. Median time to resolution of symptoms was determined from Kaplan-Meier analyses. 

Information on GSK’s data-sharing commitments and requesting access can be found at https://www.clinicalstudydatarequest.com/.

## RESULTS

### Study Population and Patient Disposition

**Figure 2.**
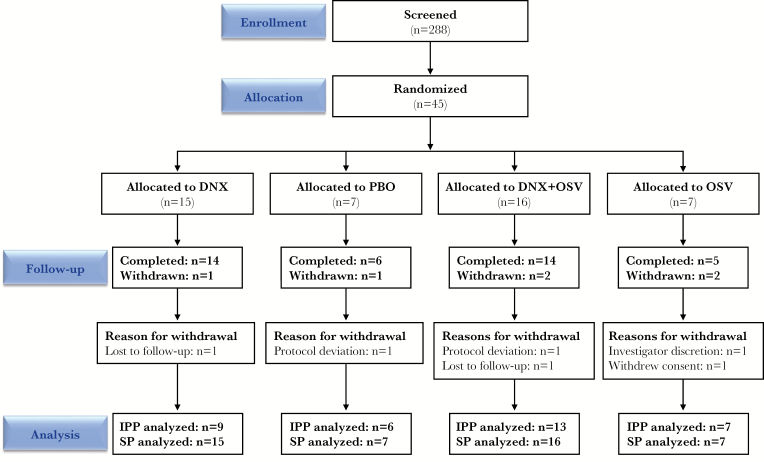
Summary of patient disposition. DNX, danirixin; IPP, influenza positive population; OSV, oseltamivir; PBO, placebo; SP, safety population.

Of 288 participants screened, 45 were enrolled, treated, and included in the safety population (Figure 2). The IPP consisted of 35 enrolled participants who were confirmed to be influenza positive by qRT-PCR or qVC at the central laboratory (Table 1). At least 70% of participants in each treatment group completed the study, including 93% (n = 14) of participants treated with DNX alone and 88% (n = 14) of those treated with DNX+OSV (Figure 2).

**Table 1.** Demographic and Baseline Characteristics (Safety Population)^a^

Characteristics	DNX (N = 15)	PBO (N = 7)	DNX+OSV (N = 16)	OSV (N = 7)	Total (N = 45)
Age, years					
Mean (SD)	37.9 (13.4)	33.4 (11.6)	42.6 (11.5)	36.3 (9.8)	38.6 (12.0)
Sex^b^					
Female	6 (40)	1 (14)	7 (44)	2 (29)	16 (36)
Male	9 (60)	6 (86)	9 (56)	5 (71)	29 (64)
Ethnicity					
Hispanic/Latino	0 (0)	0 (0)	1 (6)	1 (14)	2 (4)
Not Hispanic/Latino	15 (100)	7 (100)	15 (94)	6 (86)	43 (96)
Body mass index, kg/m^2^					
Median	28.7	29.1	27.5	31.9	29.1
Minimum	23.0	19.1	20.4	23.0	19.1
Maximum	34.6	34.0	41.4	34.2	41.4
Tobacco Use					
Never smoked	10 (67)	5 (71)	11 (69)	5 (71)	31 (69)
Current smoker	2 (13)	1 (14)	2 (13)	1 (14)	6 (13)
Former smoker	3 (20)	1 (14)	3 (19)	1 (14)	8 (18)
Race					
African American/African heritage	2 (13)	0 (0)	1 (6)	1 (14)	4 (9)
American Indian or Alaskan native	1 (7)	0 (0)	0 (0)	0 (0)	1 (2)
Asian	0 (0)	2 (29)	1 (6)	0 (0)	3 (7)
Native Hawaiian or Other Pacific Islander	1 (7)	0 (0)	0 (0)	0 (0)	1 (2)
White	11 (73)	5 (71)	13 (81)	6 (86)	35 (78)
African American/African heritage and White	0 (0)	0 (0)	1 (6)	0 (0)	1 (2)
Rapid Antigen Test					
Positive for influenza A	5 (33)	5 (71)	8 (50)	2 (29)	20 (44)
Positive for influenza B	8 (53)	2 (29)	8 (50)	5 (71)	23 (51)
Positive for influenza A and B	2 (13)	0 (0)	0 (0)	0 (0)	2 (4)
Day 1 qRT-PCR Subtype^c^					
Influenza A/H1N1	1 (7)	2 (29)	1 (6)	0 (0)	4 (9)
Influenza A/H3N2	4 (27)	2 (29)	5 (31)	2 (29)	13 (29)
Influenza B	4 (27)	2 (29)	7 (44)	5 (71)	18 (40)
Negative	6 (40)	1 (14)	2 (13)	0 (0)	9 (20)
Symptoms of Influenza					
Cough	15 (100)	7 (100)	16 (100)	7 (100)	45 (100)
Sore throat	15 (100)	7 (100)	13 (81)	7 (100)	42 (93)
Headache	14 (93)	7 (100)	16 (100)	7 (100)	44 (98)
Nasal congestion	15 (100)	7 (100)	16 (100)	7 (100)	45 (100)
Feeling feverish	14 (93)	7 (100)	16 (100)	7 (100)	44 (98)
Body aches and pains	15 (100)	6 (86)	16 (100)	7 (100)	44 (98)
Fatigue	15 (100)	7 (100)	16 (100)	7 (100)	45 (100)
Neck pain	13 (87)	6 (86)	12 (75)	5 (71)	36 (80)
Interrupted sleep	14 (93)	7 (100)	16 (100)	7 (100)	44 (98)
Loss of appetite	12 (80)	6 (86)	16 (100)	7 (100)	41 (91)

Abbreviations: DNX, danirixin; OSV, oseltamivir; PBO, placebo; qRT-PCR, quantitative reverse-transcription polymerase chain reaction; SD, standard deviation.

^a^Data are presented as n (%) unless otherwise stated.

^b^One enrolled participant was transgender.

^c^No nasopharyngeal sample was obtained for one participant in the DNX + OSV group.

The overall study population had a mean age of 39 years and was predominantly male (64%) (Table 1). The incidences of influenza symptoms were similar across the treatment groups (Table 1). Influenza subtype analysis by qRT-PCR showed a predominance of subtype B, followed by the presence of A/H3N2 and then A/H1N1; the proportions were similar across all groups (Table 1).

### Safety and Tolerability

Adverse events were experienced by 20% (n = 3) of participants in the DNX group, 44% (n = 7) of participants in the DNX+OSV group, and 57% (n = 4) in the placebo group; no participant in the OSV group experienced an AE (Table 2). Most events were Grade 2 except for one Grade 3 event (T-wave abnormality) in the DNX group (see below). Four participants treated with DNX experienced Grade 1 (dizziness) or Grade 2 (diarrhea, headache, neutropenia, and ureterolithiasis) events (Table 2) that were considered by the investigator to be drug related. Two participants prematurely discontinued treatment due to AEs; 1 participant in the DNX group experienced a Grade 3 T-wave abnormality (SAE; not treatment related), which resolved within 4 days, and 1 participant in the DNX+OSV group experienced Grade 2 treatment-related neutropenia, which resolved within 2 days. There were no reported treatment-related SAEs.

**Table 2.** AE/DRE Overview and Incidence of Specific Grade 2–4 AEs (Safety Population)

	DNX (N = 15)	PBO (N = 7)	DNX+OSV (N = 16)	OSV (N = 7)	DNX Overall (N = 31)
**Participants with any** **AE, n (%)**	3 (20)	4 (57)	7 (44)	0 (0)	10 (32)
**Any Grade 2–4 event**^**a**^	3 (20)	1 (14)	6 (38)	0 (0)	9 (29)
Back pain	0 (0)	0 (0)	1 (6)	0 (0)	1 (3)
Diarrhea	1 (7)	0 (0)	0 (0)	0 (0)	1 (3)
Ear infection bacterial^b^	1 (7)	0 (0)	0 (0)	0 (0)	1 (3)
ECG T-wave abnormal	1 (7)	0 (0)	0 (0)	0 (0)	1 (3)
Hemophilus infection^b^	1 (7)	0 (0)	0 (0)	0 (0)	1 (3)
Headache	1 (7)	0 (0)	0 (0)	0 (0)	1 (3)
Ingrown nail^b^	0 (0)	0 (0)	1 (6)	0 (0)	1 (3)
Neutrophil count decreased	0 (0)	0 (0)	1 (6)	0 (0)	1 (3)
Osteoarthritis	0 (0)	1 (14)	0 (0)	0 (0)	0 (0)
Otitis externa^b^	1 (7)	0 (0)	0 (0)	0 (0)	1 (3)
Otitis media^b^	1 (7)	0 (0)	0 (0)	0 (0)	1 (3)
Pharyngitis streptococcal	0 (0)	0 (0)	1 (6)	0 (0)	1 (3)
Tonsillitis^b^	0 (0)	0 (0)	1 (6)	0 (0)	1 (3)
Tympanic membrane perforation^b^	1 (7)	0 (0)	0 (0)	0 (0)	1 (3)
Upper RTI	0 (0)	0 (0)	1 (6)	0 (0)	1 (3)
Ureterolithiasis	1 (7)	0 (0)	0 (0)	0 (0)	1 (3)
Vomiting	0 (0)	0 (0)	1 (6)	0 (0)	1 (3)
**Any AE related to study treatment**	2 (13)	2 (29)	2 (13)	0 (0)	4 (13)
**Any AE leading to permanent discontinuation of study treatment**	1 (7)	0 (0)	1 (6)	0 (0)	2 (6)
**Participants with any SAE, n (%)**	1 (7)	0 (0)	0 (0)	0 (0)	1 (3)
**Any SAE related to study treatment**	0 (0)	0 (0)	0 (0)	0 (0)	0 (0)
**Any Fatal Event**	0 (0)	0 (0)	0 (0)	0 (0)	0 (0)
**Participants with any DRE** ^**c**^ **, n (%)**	2 (13)	0 (0)	1 (6)	0 (0)	3 (10)
Otitis media	2 (13)	0 (0)	0 (0)	0 (0)	2 (6)
Sinusitis	1 (7)	0 (0)	1 (6)	0 (0)	2 (6)
Bronchitis	0 (0)	0 (0)	1 (6)	0 (0)	1 (3)
Pneumonia	0 (0)	0 (0)	0 (0)	0 (0)	0 (0)

Abbreviations: AE, adverse event; DNX, danirixin; DRE, disease-related event; ECG, electrocardiogram; OSV, oseltamivir; PBO, placebo; RTI, respiratory tract infection; qRT-PCR, quantitative reverse-transcription polymerase chain reaction; SAE, serious adverse event.

^a^All AEs were Grade 2 except for 1 Grade 3 event (T wave abnormality) in one participant.

^b^AE occurred in one participant who was negative for influenza by qRT-PCR analysis.

^c^Per protocol, DREs of interest were not reported as AEs.

Protocol-defined DREs of interest were reported in 2 participants in the DNX group (otitis media, n = 2 and sinusitis, n = 1) (Table 2). In the DNX+OSV treatment group, 1 participant received antibiotic treatment for sinusitis. There were no cases of pneumonia reported in the study.

The only AE reported in >2 participants was vomiting, one of which was Grade 1 (DNX+OSV). There were no deaths reported in this study.

**Figure 3.**
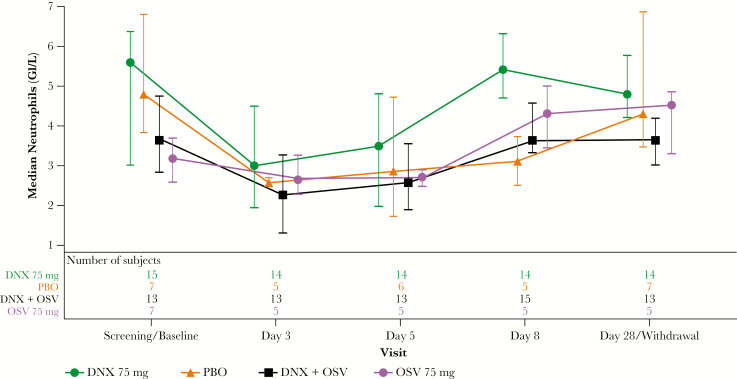
Median plot of absolute neutrophil count by visit (safety population). Bars represent interquartile ranges. DNX, danirixin; OSV, oseltamivir; PBO, placebo.

The incidences of any clinical chemistry and hematology assessments with toxicity grades of >1 were low (≤14% and ≤13% in any arm, respectively). Median neutrophil counts followed a similar pattern in all treatment groups, with decreases observed at day 3, increases by day 5 of treatment, and resolution at approximately day 8 (Figure 3). Seven participants developed transient neutropenia (2, 4, and 1 in DNX, DNX+OSV, and placebo groups, respectively), as revealed by central laboratory analyses; 1 case (in the DNX+OSV group) was reported by the investigator as an AE. There were no confirmed bacterial AEs in participants with neutropenia.

Vital signs results were similar for the DNX treatment groups compared with the placebo and OSV groups. All but 1 participant (T-wave abnormality noted above) reported normal ECGs or abnormalities that were not clinically significant. There were no safety concerns reported from the QTc analysis. Most participants, across all treatment groups, had QTc assessments of <450 milliseconds.

### Secondary and Exploratory Endpoints

#### Clinical Efficacy

Resolution of fever occurred in most participants in each treatment group during the study; time to resolution was the shortest in the DNX+OSV (median: 54 hours) and OSV (median: 76 hours) groups (Table 3). Approximately 60% of participants who had fever at screening or baseline were afebrile by day 6 across each of the 4 treatment groups. The percentage of participants who achieved resolution of all influenza symptoms (defined as the first 24-hour period in which all respiratory and systemic symptoms were absent or mild) ranged from 57% to 100% across all treatment groups (Table 3). The Kaplan-Meier analysis revealed that the DNX+OSV treatment group had the shortest time to clinical resolution of all influenza symptoms (Table 3; Supplemental Figure S1).

**Table 3.** Time to Clinical Resolution of Fever, All Influenza Symptoms, Systemic Symptoms, and Respiratory Symptoms (IPP)

	DNX (N = 9)	PBO (N = 6)	DNX+OSV (N = 13)	OSV (N = 7)
Fever				
Clinical resolution^a^ Yes, n (%)	6 (67)	6 (100)	11 (85)	5 (71)
Kaplan-Meier estimate median, hours (95% CI)	120 (15–ND)	98 (46–130)	54 (21–116)	76 (18–ND)
All Influenza Symptoms				
Clinical Resolution^a^ Yes, n (%)	6 (67)	6 (100)	12 (92)	4 (57)
Kaplan-Meier estimate median, hours (95% CI)	174 (15–ND)	147 (109–466)	112 (54–140)	267 (58–ND)
Systemic Symptoms^b^				
Clinical resolution^a^ Yes, n (%)	7 (78)	6 (100)	12 (92)	5 (71)
Kaplan-Meier estimate median, hours (95% CI)	116 (15–163)	147 (72–466)	102 (54–140)	95 (30–ND)
Respiratory Symptoms^c^				
Clinical resolution^a^ Yes, n (%)	6 (67)	6 (100)	12 (92)	4 (57)
Kaplan-Meier estimate median, hours (95% CI)	167 (15–ND)	119 (108–171)	102 (31–137)	126 (50–ND)

Abbreviations: CI, confidence interval; DNX, danirixin; Flu-iiQ, Influenza Intensity and Impact Questionnaire; IPP, influenza-positive population; ND, not determined (due to participants not achieving response by the end of the study); OSV, oseltamivir; PBO, placebo.

^a^Clinical response was missing for 2 participants due to missing baseline assessments.

^b^Systemic symptoms as defined in the Flu-iiQ: headache, feeling feverish, body aches, fatigue, neck pain, interrupted sleep, loss of appetite.

^c^Respiratory symptoms as defined in the Flu-iiQ: cough, sore throat, nasal congestion.

The percentage of participants who achieved resolution of systemic symptoms ranged from 71% to 100% across each treatment group. The DNX+OSV and OSV groups had the shortest times to resolution of systemic symptoms (Table 3). The percentage of participants who achieved resolution of respiratory symptoms ranged from 57% to 100% across each treatment group. The DNX+OSV group had the shortest time to resolution of respiratory symptoms (Table 3).

Confidence intervals for time to resolution of fever, systemic symptoms, respiratory symptoms, or all influenza symptoms were either very large and overlapping or not determined due to participants not achieving resolution by the end of the study.

#### Health Outcomes

No participants were hospitalized for influenza. However, the participant experiencing the Grade 3 SAE (T-wave abnormality) in the DNX group was admitted to hospital.

#### Virology

The viral loads, assessed by qRT-PCR, decreased in a similar manner in all treatment groups over time (Supplemental Figure S2). Similar trends were observed for qVC (data not shown). Although fewer participants in the OSV-treated groups (DNX+OSV and OSV) had detectable virus starting at day 5, there was little difference in detectable viral ribonucleic acid (RNA) between those treated with DNX (DNX and DNX+OSV) and those not receiving DNX (OSV, placebo) (Supplementary Table 2). There was no evidence of OSV resistance based on individual participant’s viral loads.

## DISCUSSION

Danirixin is hypothesized to have the greatest efficacy in hospitalized patients with complicated influenza infection; however, due to the novel approach of attenuating neutrophil infiltration during infection, the aim of this Phase IIa study was to assess the safety profile and clinical effect of DNX in otherwise healthy adults with acute, uncomplicated influenza before initiating a study in patients with complicated influenza.

A dose of DNX, 75 mg twice daily, was selected for this study because it was expected to obtain a balance between inhibition of excessive neutrophil response and maintenance of innate host defense. Models predict that 75 mg twice daily would provide 50%–60% CXCR2 antagonism over the dosing interval, which has been associated with reduced neutrophil activity and markers of lung damage in preclinical models as well as positive effects in clinical models of airway activation [18, 20, 25].

This study found that DNX treatment (with or without OSV) was well tolerated, based on the frequency of AEs, clinical chemistry evaluations, vital signs, and ECG parameters. The incidence of AEs in the treatment groups varied widely, from 0% in the OSV group to 57% in the placebo group, due to the small number of participants.

Median peripheral neutrophil counts in all treatment arms were reduced on day 3 but improved while on treatment at day 5 and resolved by day 8. Seven participants (6 in DNX treatment arms, 1 in the placebo arm) experienced neutropenia. Although several studies have shown that leukopenia is a common finding in patients with influenza, data on the incidence of neutropenia are limited. One recent study of over 400 patients suggested that approximately 15% of patients were neutropenic, with no identifiable cause other than the influenza [26]. This is in line with the observed incidence of neutropenia in the current study. Although there was an imbalance in the reports of neutrophil decreases in DNX treatment arms compared with placebo in participants with uncomplicated influenza, the resolution of the findings while continuing to receive treatment argues in favor of a virally mediated effect.

No differences in viral clearance were observed in DNX groups (with or without OSV) compared with the other treatment groups. The mean decrease in viral load from baseline, as assessed by qRT-PCR, was similar across all treatment groups. Fewer participants in the OSV-treated groups (DNX+OSV and OSV) had detectable virus starting at day 5, compared with DNX alone and the placebo group, with little difference in detectable viral RNA based on DNX treatment (Supplementary Table 2).

Danirixin is not expected to prevent viral clearance, and the dose given is not expected to lead to the development of secondary infections. Danirixin binds competitively and reversibly to the IL-8 binding site of CXCR2 with selectivity over CXCR1. Treatment with DNX is anticipated to maintain a balance between inhibiting excessive immune response, while preserving antimicrobial function by only partially reducing neutrophil recruitment, selectively inhibiting CXCR2 over CXCR1, and maintaining acquired humoral immunity and cell-mediated immunity [27–29].

Overall, there were no consistent differences in clinical outcomes (as assessed by resolution of symptoms or hospitalization due to influenza) across the 4 treatment groups. Although there was a trend towards decreased time to resolution of all symptoms in the DNX+OSV group compared with the other treatment groups, confidence intervals were very large and overlapping, in part due to the small number of participants. Therefore, interpretation of efficacy results is limited.

A limitation of this study was that fewer participants were enrolled than planned; the target enrollment for this study was 90–120 participants. However, due to mild influenza seasons and slow recruitment, enrollment was halted after 1 southern and 1 northern hemisphere influenza season. An additional limitation of this study was the presence of influenza false-positive participants; approximately 20% of participants enrolled based on positive local RATs were found to be influenza negative by central laboratory RT-PCR testing and, therefore, were not evaluable for efficacy.

## CONCLUSIONS

In conclusion, no safety concerns were identified in participants treated with DNX with or without OSV (based on frequency of AEs, clinical chemistry evaluations, vital signs, or ECG parameters). Mean neutrophil counts followed a similar trend, consistent with influenza infection, across treatment groups and viral clearance was not impacted by treatment with DNX with or without OSV. The safety results of this study enabled progression of DNX development into hospitalized patients with influenza treated with intravenous DNX [21] and may inform on future studies with this CXCR2 antagonist.

## Supplementary Data

Supplementary materials are available at *Open Forum Infectious Diseases* online. Consisting of data provided by the authors to benefit the reader, the posted materials are not copyedited and are the sole responsibility of the authors, so questions or comments should be addressed to the corresponding author.

Supplementary Material

Supplementary Figure 1

Supplementary Figure 2
