# Supplementary material for: Randomized, Double-Blind, Placebo-Controlled Study of the Safety, Tolerability, and Clinical Effect of Danirixin in Adults With Acute, Uncomplicated Influenza
Source: Open Forum Infect Dis. 2019 Apr 22;6(4):ofz072. doi: 10.1093/ofid/ofz072 (PMC6476494; doi:10.1093/ofid/ofz072)
Supplement: Supplementary Material [file ofz072_suppl_supplementary_material.docx]

**SUPPLEMENTARY MATERIAL**

**Study Inclusion Criteria**

Patients were included in the study if they were 18–64 years of age (inclusive) at the time of consent; had a body weight of >60 kg for men and >45 kg for women and a body mass index between 19 kg/m^2^ and 35 kg/m^2^, inclusive; had experienced the onset of influenza-like illness symptoms within 48 hours prior to study enrollment (onset of symptoms was defined as the time when the patient’s temperature was measured as elevated (≥38.0°C [≥100.4°F]) or the time when the patient first experienced at least one symptom [cough, sore throat, nasal congestion, headache, feeling feverish, body aches and pains, or fatigue]); had an oral temperature (≥38.0°C; ≥100.4°F) at the screening visit or history of feeling feverish within the 24 hours prior to screening visit; had at least one respiratory symptom (cough, sore throat, nasal congestion) and at least one systemic symptom (headache, body aches and pain, fatigue) due to influenza infection; and had a positive antigen test. Male patients with female partners of childbearing potential were eligible for inclusion if their partners complied with the following contraception requirements from the time of first dose of study medication until at least 36 hours (five half-lives) after the last dose of study medication: vasectomy with documentation of azoospermia; male condom, and the partner must use of one of the contraceptive options: contraceptive subdermal implant; intrauterine device or intrauterine system; oral contraceptive, either combined or progestogen alone; injectable progestogen; contraceptive vaginal ring; or percutaneous contraceptive patches. Female patients were eligible to participate if they were not pregnant (as confirmed by a negative urine human chorionic gonadotrophin test), not lactating, and at least one of the following conditions applied: non-reproductive potential defined as either pre-menopausal with: documented tubal ligation; documented hysteroscopic tubal occlusion procedure with follow-up confirmation of bilateral tubal occlusion; hysterectomy; and documented Bilateral Oophorectomy; or postmenopausal (defined as 12 months of spontaneous amenorrhea). Females on hormone replacement therapy whose menopausal status was in doubt were required to use one of the previously defined highly effective contraception methods if they wished to continue hormone replacement therapy. Patients also had to be willing and able to give written informed consent to participate in the study and to adhere to the procedures stated in the protocol.

**Study Exclusion Criteria**

Patients were excluded from the study if they were defined as being at high risk of complications from influenza infection (according to the World Health Organization Guidelines for Pharmacological Management of Pandemic Influenza A [H1N1] and other influenza viruses), including: pregnant women; and persons with chronic pulmonary, cardiac, renal or hepatic disease, metabolic disorder, certain neurological conditions (including neuromuscular, neurocognitive, and seizure disorders), or hemoglobinopathies or immunosuppression (primary immunosuppressive condition such as HIV, or secondary to immunosuppressive medication or malignancy). Patients were also excluded if they met any of the following criteria: severe illness anticipated to require in-hospital care; QTc >450 msec or QTc >480 msec in participants with Bundle Branch Block; history of sensitivity to any of the study medications or components thereof; had taken an approved or investigational anti-influenza medication within the last 4 weeks prior to enrollment; had received the live attenuated influenza virus vaccine within 21 days prior to the study; required treatment with an influenza antiviral that was considered essential; use of oral or injectable CYP3A4 or BCRP substrates with a narrow therapeutic index, or oral or systemic glucocorticoids, during the study period; systemic steroids or immunosuppressants within the previous 2 weeks; oral or systemic antibiotics within 1 week prior to enrollment; suspected or confirmed bacterial infection; experiencing complications of respiratory tract infection, showing signs of severe or progressive disease, or worsening of any pre-existing medical condition at the time of enrollment, considered by the investigator to place the participant at unreasonably increased risk from participation in the study; history of alcohol/drug abuse within
6 months of the study start date; consumption of >3 alcoholic units within 24 hours prior to study entry; pregnancy; breastfeeding.

**Supplementary Table 1. Investigator Sites That Enrolled Participants Into This Study**

| **Investigator** | **Description of Research Facility/Institution, and Address** |
| --- | --- |
| Bangash, Mohammad AK. MBBS | Liverpool Family Medical Centre, 279 Macquarie Street, Liverpool, New South Wales, 2170, Australia |
| Blom, Hans A. MBBS, RACOG | Vale Medical Practice, Brookvale House, 1A Cross Street, Brookvale, New South Wales 2100, Australia |
| Brijmohan, Gibraun. MBBS, FRACGP | Fountain Valley Medical Centre, 2 Alabama Road, Happy Valley, South Australia, 5159, Australia |
| Cohen, Isaac S. MBChB, FRACGP | Pakenham Superclinic, 1 LyleBlue Court, Pakenham, Victoria, 3180, Australia |
| Islam, Nazrul MD. MBBS, FRACGP | Wyndham Village Medical Centre, Wyndham Village Shopping Centre, 380 Sayers Road, Tameit, Victoria, 3029, Australia |
| Jeong, James S. MBBS, FRACGP | Railway Street Medical Centre, 7-11 Railway Street, Baulkham Hills, New South Wales, 2153, Australia |
| Karthigesu, Dhanlakshimi. MBBS, FRACGP | Valley Plaza Medical Centre, Shop 5B Valley Plaza, 189 Wilson Road, Hinchinbrook, New South Wales, 2168, Australia |
| Kerwin, Edward Michael. MD | Clinical Research Institute of Southern Oregon, PC, Suite B, 3860 Crater Lake Avenue, Medford, Oregon, 97504, USA |
| Kwong, Kin Chung William. MBChB, FRACGP | Springfield Superclinic, 29-31 Commercial Drive, Springfield, Queensland, 4300, Australia |
| Mitha, Essack. MBChB | Newtown Clinical Research Centre, 104 Jeppe Street, Johannesburg, Gauteng, 2113, South Africa |
| O'Malley-Ford, Judith M. MBBS, FRACGP | Kedron Park 7 Day Medical Centre, Corner 136 Gympie and Brookfield Roads, Kedron, Queensland, 4031, Australia |
| Rillstone, Dominic J. FRACGP | Casey Superclinic, 50 Kangan Drive, Berwick, Victoria, 3806, Australia |
| Rupasinghe, Shamnika D. MBChB, FRACGP | Lynbrook Village Medical Centre, Shop 1, Lynbrook Boulevard, Lynbrook, Victoria, 3975, Australia |
| Soucie, Gary Wayne. MD | Elite Clinical Trials, Suite T-8, 1443 Parkway Drive, Blackfoot, Idaho, 83221, USA |
| Tarpay, Martha M. MD | Allergy, Asthma and Clinical Research Center, Suite 206, 4200 West Memorial Road, Oklahoma City, Oklahoma, 73120, USA |
| Taylor, Andrew T. MBBS | Applecross Medical Group, 764 Canning Highway, Applecross, Western Australia, 6153, Australia |
| Wilhase, Agatha Cathrine. MBBCh | Practice [Wilhase], 0 Arthur Hobbs Street, Reiger park, Boksburg, Gauteng, 1459, South Africa |

**Supplementary Table 2. Participants With No Detectable Influenza Viral RNA by qRT-PCR From Nasopharyngeal Swabs Over Time (IPP)**

|  | **DNX (N=9)** | **PBO (N=6)** | **DNX+OSV (N=13)** | **OSV (N=7)** | **Total DNX**  **(N=22)** | **Total  non-DNX (N=13)** |
| --- | --- | --- | --- | --- | --- | --- |
| **Screening/baseline** |  |  |  |  |  |  |
| **n**  **Virus not detected, n (%)** | 9  0 | 6  0 | 13  0 | 7  0 | 22  0 | 13  0 |
| **Day 3** |  |  |  |  |  |  |
| **n**  **Virus not detected, n (%)** | 9  0 | 6  0 | 12  0 | 5  0 | 21  0 | 11  0 |
| **Day 5** |  |  |  |  |  |  |
| **n**  **Virus not detected, n (%)** | 9  0 | 6  0 | 12  3 (25) | 5  1 (20) | 21  3 (14) | 11  1 (9) |
| **Day 8** |  |  |  |  |  |  |
| **n**  **Virus not detected, n (%)** | 8  3 (38) | 6  1 (17) | 12  6 (50) | 5  5 (100) | 20  9 (45) | 11  6 (55) |
| **Day 14** |  |  |  |  |  |  |
| **n**  **Virus not detected, n (%)** | 9  5 (56) | 5  4 (80) | 12  11 (92) | 5  5 (100) | 21  16 (76) | 10  9 (90) |

Abbreviations: DNX, danirixin; IPP, influenza positive population; OSV, oseltamivir; PBO, placebo; qRT-PCR, quantitative reverse transcription polymerase chain reaction; RNA, ribonucleic acid

**Supplemental Figure S1. Kaplan−Meier Analysis of Time to Clinical Resolution of Illness (IPP)**

DNX, danirixin; IPP, influenza positive population; OSV, oseltamivir; PBO, placebo.

**Supplementary Figure S2. Mean Change From Baseline in Influenza Viral Load (qRT-PCR) From Nasopharyngeal Swabs (IPP)**

DNX, danirixin; IPP, influenza positive population; OSV, oseltamivir; PBO, placebo; qRT-PCR, quantitative reverse transcription polymerase chain reaction.
