# Supplementary figures and images for: Randomized, Double-Blind, Placebo-Controlled Study of the Safety, Tolerability, and Clinical Effect of Danirixin in Adults With Acute, Uncomplicated Influenza
Source: Open Forum Infect Dis. 2019 Apr 22;6(4):ofz072. doi: 10.1093/ofid/ofz072 (PMC6476494; doi:10.1093/ofid/ofz072)

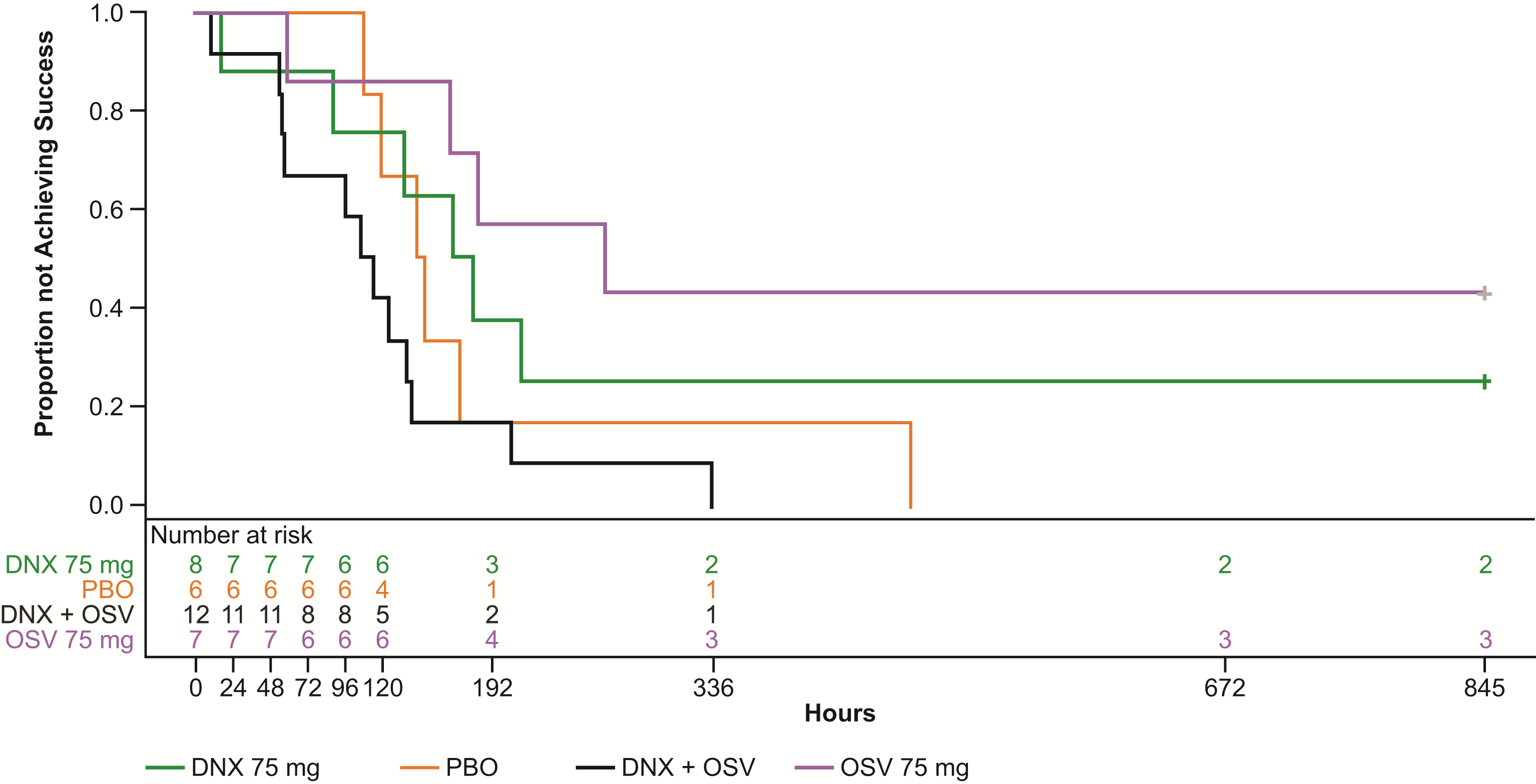

Supplement: Supplementary Figure 1 [file ofz072_suppl_supplementary_figure-1.png]

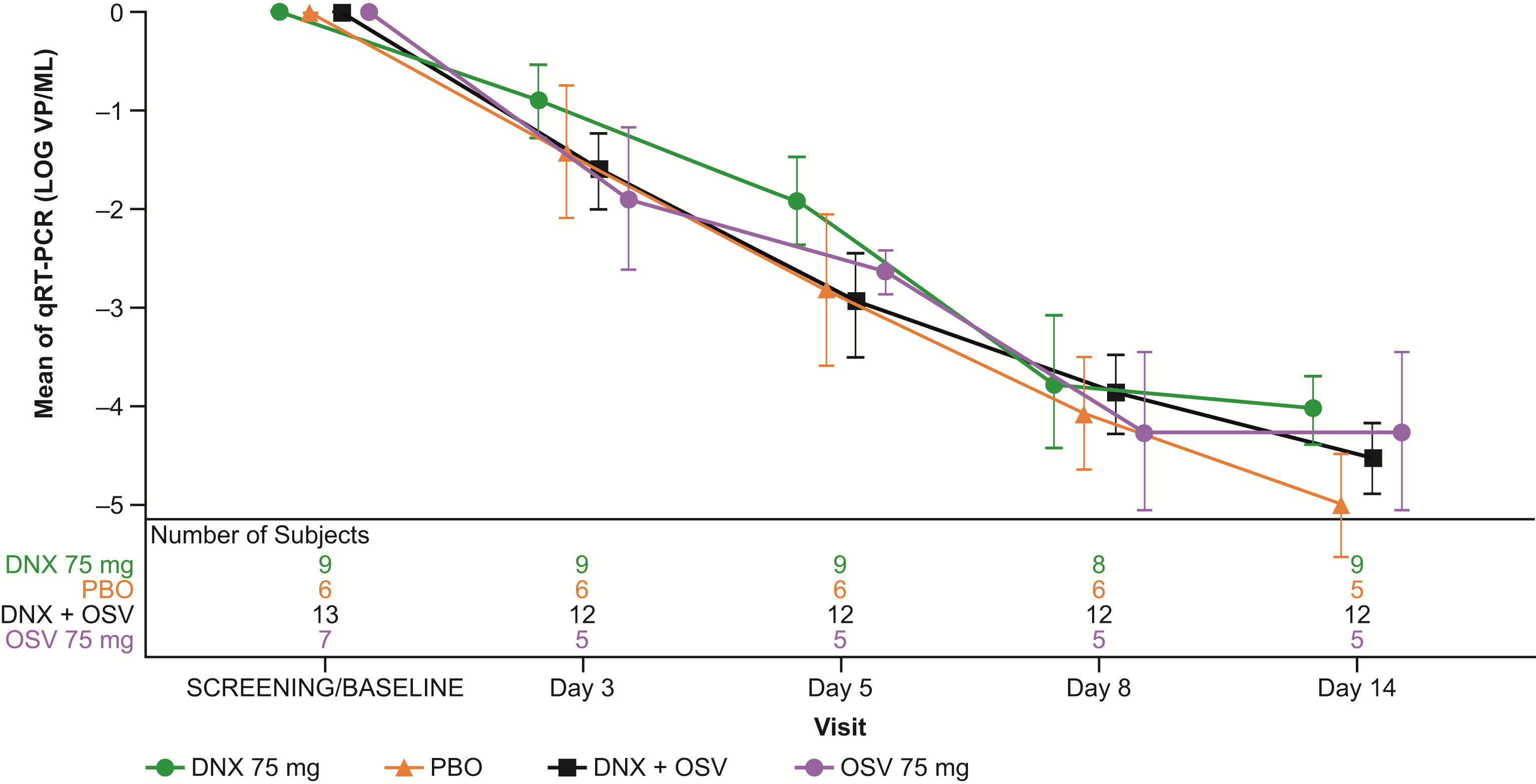

Supplement: Supplementary Figure 2 [file ofz072_suppl_supplementary_figure-2.png]
